# Interferon-γ induces immunosuppression in salivary adenoid cystic carcinoma by regulating programmed death ligand 1 secretion

**DOI:** 10.1038/s41368-022-00197-x

**Published:** 2022-09-28

**Authors:** Qiuyun Fu, Xingchi Liu, Houfu Xia, Yicun Li, Zili Yu, Bing Liu, Xuepeng Xiong, Gang Chen

**Affiliations:** 1 The State Key Laboratory Breeding Base of Basic Science of Stomatology & Key Laboratory of Oral Biomedicine Ministry of Education, School and Hospital of Stomatology, Wuhan University, Wuhan, China; 2 Department of Oral and Maxillofacial Surgery, School and Hospital of Stomatology, Wuhan University, Wuhan, China; 3 Peking University Shenzhen Hospital, Shenzhen Peking University-The Hong Kong University of Science and Technology Medical Center, Shenzhen, China; 4 Frontier Science Center for Immunology and Metabolism, Wuhan University, Wuhan, China

**Keywords:** Oral cancer, Cell biology

## Abstract

Interferon-γ (IFN-γ), a key effector molecule in anti-tumor immune response, has been well documented to correlate with the intratumoral infiltration of immune cells. Of interest, however, a high level of IFN-γ has been reported in salivary adenoid cystic carcinoma (SACC), which is actually a type of immunologically cold cancer with few infiltrated immune cells. Investigating the functional significance of IFN-γ in SACC would help to explain such a paradoxical phenomenon. In the present study, we revealed that, compared to oral squamous cell carcinoma cells (a type of immunologically hot cancer), SACC cells were less sensitive to the growth-inhibition effect of IFN-γ. Moreover, the migration and invasion abilities of SACC cells were obviously enhanced upon IFN-γ treatment. In addition, our results revealed that exposure to IFN-γ significantly up-regulated the level of programmed death ligand 1 (PD-L1) on SACC cell-derived small extracellular vesicles (sEVs), which subsequently induced the apoptosis of CD8^+^ T cells through antagonizing PD-1. Importantly, it was also found that SACC patients with higher levels of plasma IFN-γ also had higher levels of circulating sEVs that carried PD-L1 on their surface. Our study unveils a mechanism that IFN-γ induces immunosuppression in SACC via sEV PD-L1, which would account for the scarce immune cell infiltration and insensitivity to immunotherapy.

## Introduction

Salivary adenoid cystic carcinoma (SACC) is a common salivary gland malignancy and characterized by low immunogenicity.^1,2^ The 10-year survival rate of SACC patients is 50%. However, only 10% of the patients can survive more than 10 years with distant metastasis.^3,4^ Treatment strategies for SACC have changed little over the past decades, and no standard therapy is currently being indicated for the patients with recurrence and distant metastasis.^5,6^ In recent years, immunotherapy using programmed death ligand 1/programmed death 1(PD-L1/PD-1) inhibitors has shown efficacy in multiple cancer types.^7,8^ Disappointingly, SACC patients showed incidental responses to anti-PD-1 immunotherapy.^8–11^

Interferon-γ (IFN-γ) is a major effector molecule implicated in anti-tumor immune response, capable of regulating the major histocompatibility complex molecules on immunocytes and tumor cells, thus making tumor cells more susceptible to immune recognition.^12–14^ IFN-γ also stimulates the production of chemokines to promote T cell infiltration and to induce tumor apoptosis.^15,16^ Of interest, it was reported that the plasma IFN-γ levels of SACC patients was much higher than that of patients with oral squamous cell carcinoma (OSCC), which is a type of immunologically hot cancer.^17^ But conversely, in most cases, SACC were infiltrated by low densities of CD8^+^ T cells and antigen-presenting cells, whereas OSCC exhibited the opposite trend.^2^ Furthermore, SACC patients with higher levels of plasma IFN-γ also tend to have higher risks of distant metastasis. The mechanisms underneath these seemingly conflicting phenomena shall shed light on the few immunocyte infiltrations and distant metastasis of SACC.

PD-L1 on tumor cell surface binds PD-1 on T cells to elicit immune checkpoint responses, which assist tumor cells in evading immune surveillance.^7,18^ Small extracellular vesicles (sEVs, diameter < 200 nm), including exosomes and small microvesicles, are membrane vesicles carrying bioactive molecules that orchestrate extracellular communication.^19–21^ Our previous work has emphasized the key effect of IFN-γ in the up-regulation of PD-L1 on tumor-derived exosomes, which suppresses T cell responses through PD-L1/PD-1 axis.^22^ To date, however, the biological function of sEVs, secreted by SACC cells upon IFN-γ stimulation, remains largely elusive.

In this study, we found that, compared to OSCC cells, SACC cells were more insensitive to the inhibitory effects induced by IFN-γ. Moreover, IFN-γ significantly enhanced the migration and invasion abilities of SACC cells. In addition, IFN-γ also promoted the secretion of sEV PD-L1 by SACC cells. Consistently, in the plasma of SACC patients, the level of circulating sEV PD-L1 positively correlates with that of IFN-γ, and these circulating sEV PD-L1 exhibits pro-apoptosis effects by interacting with PD-1 on CD8^+^ T cells. These data collectively contribute to sketch a mechanism how does IFN-γ induce immune escape of SACC, which would account for the scarce immune cell infiltration and insensitivity to immunotherapy.

## Results

### SACC is insensitive to IFN-γ-induced PD-L1-upregulating effect and proliferation-limiting effect

**Fig. 1:**
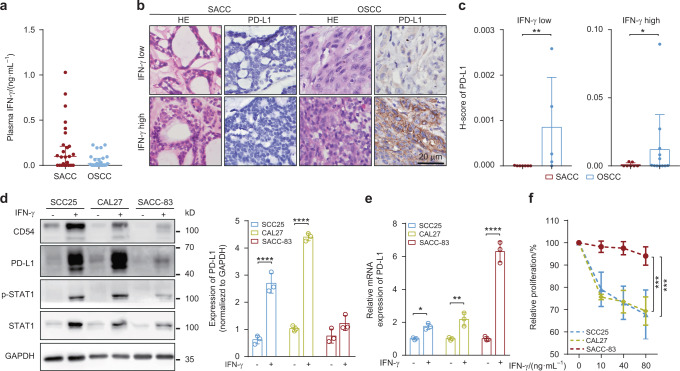
Quantification of plasma IFN-γ in SACC and OSCC patients and comparison the sensitivity of SACC cells and OSCC cells to IFN-γ. **a** Plasma IFN-γ levels were determined by ELISA in SACC samples (*n* = 25) and OSCC samples (*n* = 23) (*P* = 0.058 6). **b** Representative hematoxylin and eosin stained images and expression levels of PD-L1 in SACC (*n* = 14) and OSCC samples (*n* = 17) were determined by immunohistochemical staining. Patients with undetectable plasma IFN-γ were included in IFN-γ low group and those with detectable plasma IFN-γ were included in IFN-γ high group. Scale bar: 20 μm. **c** H-score of PD-L1 of SACC and OSCC samples. **d** Western blot analysis of IFN-γ/STAT1/PD-L1 pathway on SCC25, CAL27, and SACC-83 with or without IFN-γ treatment for 18 h (left, *n* = 3). Quantification of the western blotting data (right, normalized to GAPDH, *n* = 3). **e** mRNA expression of PD-L1 in SCC25, CAL27, and SACC-83 upon IFN-γ treatment for 12 h (*n* = 3). **f** CCK8 assay showing the effect of IFN-γ treatment on proliferation of CAL27, SCC25, and SACC-83 for 48 h (*n* = 3). *, *P* < 0.05; **, *P* < 0.01; ***, *P* < 0.001; ****, *P* < 0.000 1

Plasma IFN-γ levels of SACC patients (*n* = 25) and OSCC patients (*n* = 23) were measured by ELISA (demographic data and clinicopathological features were shown in Appendix table 1, 2). Specifically, SACC patients had a higher level of IFN-γ than OSCC ones ((0.203 1 ± 0.275) ng·mL^−1^ vs (0.057 4 ± 0.078) ng·mL^−1^) (Fig. 1a). Presented IHC staining showed that, in both low and high plasma IFN-γ groups, the expression levels of PD-L1 in SACC patients were lower than those in OSCC patients (Fig. 1b, c). Weaker activation of IFN-γ/STAT1/PD-L1 pathway was found in SACC-83 than in SCC25 and CAL27 (Fig. 1d). However, upon IFN-γ stimulation, the transcriptional level of PD-L1 in SACC-83 was significantly higher than that of SCC25 and CAL27 (Fig. 1e). IFN-γ induced trafficking of PD-L1 in SACC cells maybe related to their immune escape. In addition, CCK8 assay was also performed to confirm the insensitivity of SACC-83 to IFN-γ. These results showed the proliferation of SACC-83 was less affected by IFN-γ, whereas the proliferation of CAL27 and SCC25 decreased significantly upon IFN-γ treatment (Fig. 1f).

### IFN-γ promotes the migration and invasion of SACC through epithelial-mesenchymal transition

**Fig. 2:**
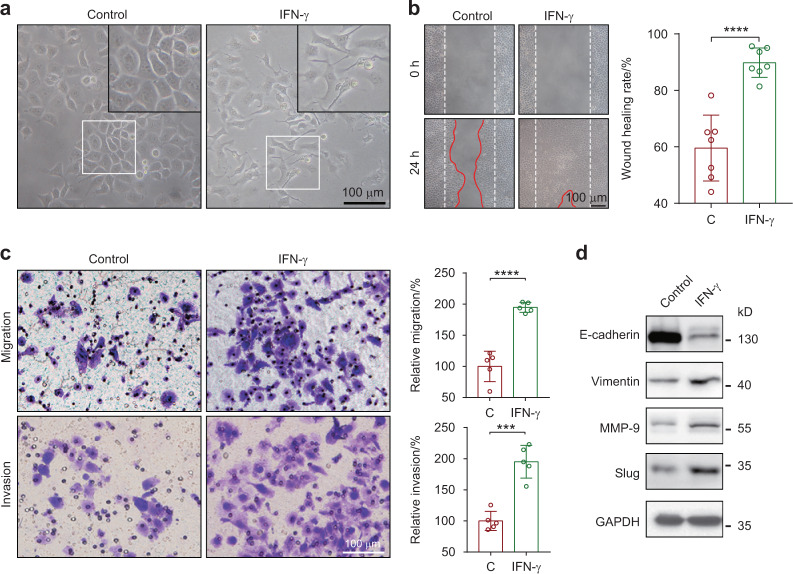
Effects of IFN-γ on migration and invasion of SACC-83. **a** Representative images showing the morphological changes of SACC-83 upon IFN-γ treatment for 48 h. **b** Wound healing assay of SACC-83 upon IFN-γ treatment for 24 h. Control group (C) and IFN-γ-treated group (IFN-γ) were quantified by Image J. Wound size was presented as the wound healing rate (*n* = 7). **c** The migration and invasion of SACC-83 upon IFN-γ treatment for 18 h were determined by transwell assay. The numbers of invading cells of IFN-γ-treated group (IFN-γ) were normalized to the control group (C) (*n* = 5). **d** Western blot analysis of EMT-related proteins. ***, *P* < 0.001; ****, *P* < 0.000 1; scale bar: 100 μm

Plasma IFN-γ levels correlate with distant metastasis in SACC patients (Appendix Table 2). Upon IFN-γ treatment, we found SACC-83 cells exhibited multiantenna morphology (Fig. 2a) and significantly enhanced migration and invasion abilities (Fig. 2b, c). Previous studies highlighted the role of epithelial-mesenchymal transition (EMT), characterized by down-regulated expression of E-cadherin and up-regulated expression of Vimentin, matrix metalloproteinases (MMPs), and Slug, in tumor migration and invasion.^23–25^ Our results showed that IFN-γ promotes the EMT of SACC-83, characterized by downregulation of E-cadherin, and upregulation of Vimentin, Slug and MMP9 (Fig. 2d). In addition, we found that SACC-LM, a cell line prone to lung metastasis, was more insensitive to IFN-γ than SACC-83 (Appendix Fig. 1a). Upon IFN-γ treatment, SACC-LM exhibited less susceptible in apoptosis and proliferation (Appendix Fig. 1b, c).

### IFN-γ promotes the PD-L1^+^ sEV secretion of SACC

**Fig. 3:**
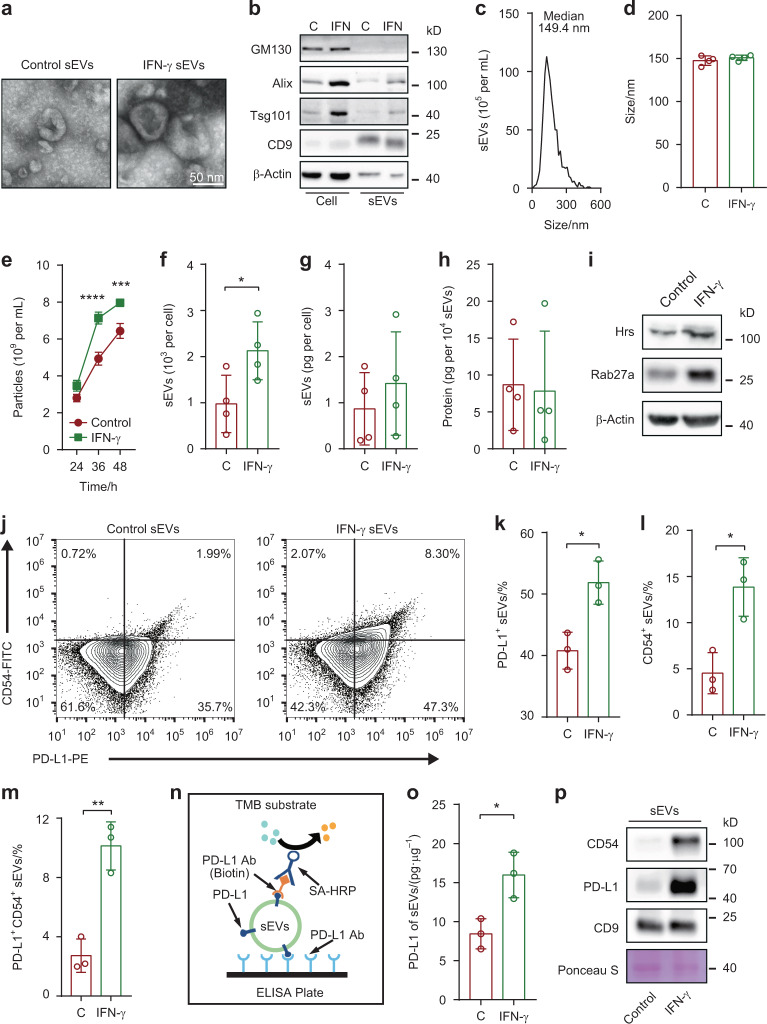
Effects of IFN-γ on sEVs derived by SACC-83. **a** Representative TEM images of the purified control sEVs and IFN-γ-induced sEVs secreted by SACC-83. Scale bar: 50 nm. **b** Western blot analysis indicating the presence of the EV makers CD9, Alix and Tsg101, and the absence of the negative marker GM130 in the purified sEVs. **c** Characterization of the purified sEVs by NTA. **d** Comparison of the sizes between control sEVs (C) and IFN-γ-induced sEVs (IFN-γ) derived by SACC-83 (*n* = 4). **e** Curves of the sEV secretion of SACC-83 cells upon IFN-γ treatment (*n* = 3). **f** Comparison of the numbers of sEVs between control group (C) and IFN-γ treatment group (IFN-γ) derived by SACC-83 (*n* = 4). **g**, **h** Comparison of the protein contents of sEVs between control group (C) and IFN-γ treatment group (IFN-γ) (*n* = 4). **i** Western blot analysis of Rab27a and Hrs in the control cells and IFN-γ treated cells (IFN-γ). **j** Representative contour plots of the control sEVs and IFN-γ-induced sEVs. **k**–**m** Comparisons of the ratios of PD-L1^+^, CD54^+^ and PD-L1^+^CD54^+^ sEVs between control sEVs (C) and IFN-γ-induced sEVs (IFN-γ) respectively (*n* = 3). **n** Diagram of quantification of sEV PD-L1 by ELISA. **o** sEV PD-L1 were measured by ELISA (*n* = 3). **p** Western blot analysis of CD54 and PD-L1 in the control sEVs and IFN-γ-induced sEVs. *, *P* < 0.05; **, *P* < 0.01; ***, *P* < 0.001; ****, *P* < 0.000 1

Our previous study identified that sEVs are important carriers for PD-L1, which elicits immune checkpoint response by interacting with PD-1 on T cells to promote immunosuppression.^22^ To determine whether SACC cells transport PD-L1 onto sEVs upon IFN-γ treatment, SACC-83 cell-derived sEVs were isolated by ultracentrifugation and confirmed by transmission electron microscopy (TEM), western bolt analysis, and nanoparticle tracking analyzer (NTA) (Fig. 3a-c). No obvious difference in sEV sizes was found (Fig. 3d), whereas elevated sEV secretion level of SACC-83 upon IFN-γ treatment was determined by NTA (Fig. 3e, f) and BCA protein assay (Fig. 3g). No difference was found in total protein content on these sEVs (Fig. 3h). Our previous study had demonstrated that endosomal sorting complex required for transport (ESCRT) subunit Hrs plays a critical role in regulating exosomal PD-L1 secretion.^22^ Rab27a, a key molecule that promotes the fusion of multivesicular bodies (MVB), boosts the secretion of EVs.^26–28^ Thus, Hrs and Rab27a were upregulated in SACC-83 upon IFN-γ treatment (Fig. 3i).

CD54 (intercellular cell adhesion molecule-1, ICAM-1), the major ligand for LFA-1 (CD18), is of paramount importance to intercellular adhesion.^29^ Nano-flow cytometry was applied for quantifying the expression levels of PD-L1 and CD54 on sEV surface, and the gating strategies were shown in Appendix Fig. 2a. Compared with control sEVs (Fig. 3j left panel), the expression of CD54 and PD-L1 on IFN-γ-induced sEVs increased significantly (Fig. 3j right panel, Fig. 3k-m, Appendix Fig. 2b, c). ELISA was used to detect PD-L1 on sEVs following our previous protocol (Fig. 3n), and the results showed significantly higher PD-L1 proteins on IFN-γ-induced sEVs than on control sEVs (Fig. 3o). Finally, the results of western bolts reconfirmed the upregulation of CD54 and PD-L1 on IFN-γ-induced sEVs (Fig. 3p).

### sEV PD-L1 promotes T cell apoptosis

**Fig. 4:**
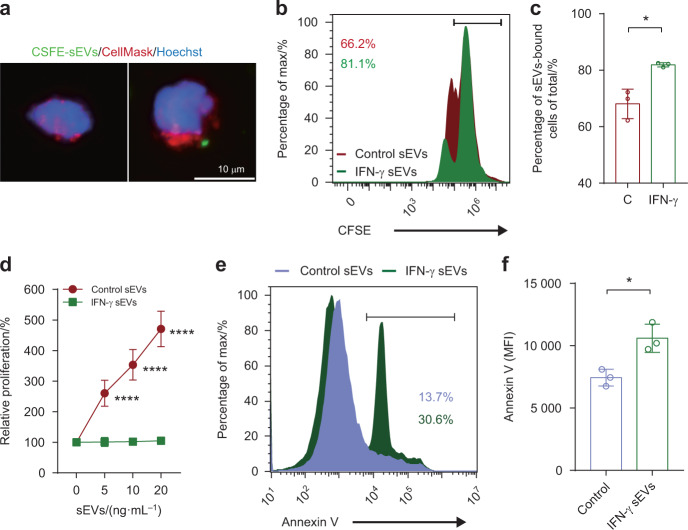
Effects of IFN-γ-induced sEVs derived from SACC-83 on T cells. **a** Immunofluorescence staining of CellMask-labeled Jurkat T cells (stimulated with PMA + Ion) after incubation with CFSE-labeled sEVs derived from SACC-83 for 12 h. Scale bar: 10 μm. **b** Representative histogram of Jurkat T cells with PMA + Ion stimulation after incubating with CFSE-labeled control sEVs and IFN-γ-induced sEVs for 12 h. **c** Comparison of the proportion of sEVs-bound cells (CFSE positive) between control sEVs (C) and IFN-γ-induced sEVs (IFN-γ) (*n* = 3). **d** CCK8 assay showing the relative proliferation of Jurkat T cells with PMA + Ion stimulation after incubation with control sEVs and IFN-γ-induced sEVs for 24 h (*n* = 4). **e** Representative histogram of Jurkat T cells with PMA + Ion stimulation after incubating with IFN-γ-induced sEVs for 12 h. **f** The mean fluorescence intensity (MFI) of Annexin V of Jurkat T cells after incubation with IFN-γ-induced sEVs (*n* = 3). *, *P* < 0.05; ****, *P* < 0.000 1

We also studied the functions of these sEVs. To explore the roles of IFN-γ-induced sEVs to immune cells, we activated and co-incubated Jurkat T cells with sEVs. Immunofluorescence staining showed that CFSE-labeled sEVs could bind to activated Jurkat T cells (Fig. 4a). Upon activation via PMA and Ion, Jurkat T cells upregulated surface PD-1 expression (Appendix Fig. 3a). After co-incubation of these Jurkat T cells with CFSE-labled control sEVs and IFN-γ-induced sEVs, Jurkat T cells showed enhanced binding with IFN-γ-induced sEVs (Fig. 4b, c, Appendix Fig. 3b, c). Anti-CD18 antibody blocked the increasing trend of the binding of IFN-γ-induced sEVs (Appendix Fig. 3b, c).

We then tested the role of these sEVs on the proliferation of Jurkat T cells, and found that IFN-γ-induced sEVs significantly suppressed the proliferation of Jurkat T cells (Fig. 4d). IFN-γ-induced sEVs promoted the apoptosis of Jurkat T cells (Fig. 4e, f), and this pro-apoptotic effect was dependent on sEV PD-L1 (Appendix Fig. 3d). After incubating Jurkat T cells with anti-PD-1 antibody, the pro-apoptotic effect of IFN-γ-induced sEVs was decreased (Appendix Fig. 3d).

In addition, we validated the role of IFN-γ-induced sEVs in human primary immunocytes. Primary immunocytes were activated with PMA + Ion before co-incubation with IFN-γ-induced sEVs (Appendix Fig. 3e), and CD8^+^ T cells were gated as depicted in Appendix Fig. 3f. The results showed that IFN-γ-induced sEVs exhibited pro-apoptotic effect on CD8^+^ T cells, while pre-treating the cells with anti-PD-1 blocking antibody attenuated the pro-apoptotic effect of IFN-γ-induced sEVs (Appendix Fig. 3g, h). These data suggest that sEV PD-L1 promotes the apoptosis of T cells.

### Circulating sEV PD-L1 promotes the apoptosis of primary CD8^+^ T cells

**Fig. 5:**
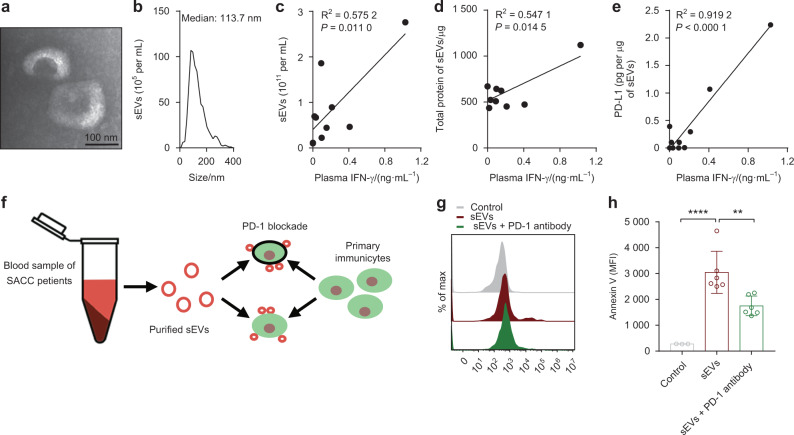
Correlations between circulating sEVs with plasma IFN-γ levels and the effects of circulating sEVs on human CD8^+^ T cells. **a** Representative TEM images of the isolated circulating sEVs. Scale bar: 100 nm. **b** Characterization of the isolated circulating sEVs by NTA. **c** Correlation analysis between the NTA-measured concentration levels of circulating sEVs and plasma IFN-γ level (*n* = 10). **d** Correlation analysis between the total protein contents of sEVs purified from 500 μL plasma and plasma IFN-γ level (*n* = 10). **e** Correlation analysis between protein content of PD-L1 on circulating sEVs and plasma IFN-γ level (*n* = 10). **f** Diagram of incubation circulating sEVs from the SACC patients with primary immunocytes. **g** Representative histogram of CD8^+^ T cells with PMA + Ion stimulation after incubation with circulating sEVs for 12 h. **h** The MFI of Annexin V of CD8^+^ T cells after incubating with circulating sEVs with or without anti PD-1antibody blocking (*n* = 6). **, *P* < 0.01; ****, *P* < 0.000 1

We purified circulating sEVs from SACC patients (*n* = 10), and then identified these vesicles by TEM and NTA (Fig. 5a, b). Interestingly, the number and total protein levels of these circulating sEVs correlated with the plasma IFN-γ levels (Fig. 5c, d). PD-L1 on these circulating sEVs measured by ELISA also correlated with the plasma IFN-γ levels (Fig. 5e). Circulating sEVs with high PD-L1 level (*n* = 6, > 0.1 pg per μg of sEVs) were selected for further experiment. Following the flow chart, we determined the effect of circulating sEV PD-L1 on primary immunocytes (Fig. 5f). First, primary immunocytes were activated with PMA + Ion before co-incubation with circulating sEVs, and CD8^+^ T cells were identified as mentioned in Appendix Fig. 3f. Flow cytometry analyses indicated that circulating sEV PD-L1 increased the level of Annexin V on CD8^+^ T cells, and this increase could be reduced by anti-PD-1 antibody pretreatment (Fig. 5g, h). These data indicate that circulating sEV PD-L1 mediates CD8^+^ T cell apoptosis.

## Discussion

Weekly systemic administration of IFN-γ increased the infiltration of T lymphocytes in cold tumors,^30^ and intratumoral injection of IFN-γ increased the level of CXCL10 and CXCL11, cytokines promoting intratumoral immune cell infiltration, in melanoma patients.^31,32^ However, SACC patients have a higher level of plasma IFN-γ but scarce tumor-infiltrating lymphocytes (TIL). In this study, we found SACC cells, upon IFN-γ treatment, enhanced their migration and invasion rather than inhibited their growth. Also, in response to IFN-γ, SACC cells synthesized more PD-L1 and secreted them extracellularly via sEVs instead of keeping them inside cells, and these sEVs-associated PD-L1 induced T cell apoptosis through interacting with its surface PD-1 (Appendix Fig. 4). This work explained the plausibility that the low density of TIL occurs in a high IFN-γ circumstance of SACC patients.

In the present study, SACC patients have higher plasma IFN-γ levels than those with OSCC, an immunologically hot tumor enrolled here as control, which is consistent with a previous study.^17^ In contrast to OSCC, SACC expressed lower level of cellular PD-L1. We then further disclosed in vitro, using human recombinant IFN-γ as stimulus, SACC-83 cells exhibited weaker activation of IFN-γ/STAT1/PD-L1 pathway than SCC25 and CAL27 cells, which explained the negative expression of PD-L1 in paraffin sections of SACC. On the other hand, upon IFN-γ stimulation, the decreasing trend of the proliferation of SACC-83 was less than those of SCC25 and CAL27. These results suggested SACC is IFN-γ insensitive, which may account for the few immune cells and antigen-presenting cells infiltrations in SACC.^33,34^ In addition, β-catenin/Wnt and PI3K pathways had been reported to be related with the lack of TIL in SACC.^35^ Of note, the source of plasma IFN-γ in SACC patients remains elusive and needs further investigation.

We found that the SACC patients with high plasma IFN-γ level were apt to develop distant metastatic disease. As expected, IFN-γ significantly increased the migratory and invasive capabilities of SACC-83, and the similar effects have been documented in pancreatic cancer, gastric cancer, and melanoma.^36–38^ We then compared the responses of SACC-83 with SACC-LM upon IFN-γ treatment, and found SACC-LM exhibited a greater insensitivity in IFN-γ/STAT1/PD-L1 pathway activation, proliferation inhibition, and apoptosis induction. Nevertheless, whether SACC cells with enhanced migratory and invasive capabilities educated by IFN-γ would colonize lung needs further demonstration. Paradoxically, IFN-γ significantly enhanced the mRNA level of PD-L1 in SACC, whereas its protein level was only mildly elevated. To understand the trafficking of PD-L1 in SACC upon IFN-γ treatment, we measured the PD-L1 expression levels on sEVs. Of interest, IFN-γ stimulated SACC-83 cells secreted more PD-L1-positive sEVs. Also, PD-L1 on those sEVs promoted the apoptosis of Jurkat T cells and primary CD8^+^ T cells. Moreover, IFN-γ upregulated the expression of CD54 on those sEVs, which enhanced the uptaking of sEV by Jurkat T cells via binding LFA-1 (CD18). These findings suggest that rather than keeping PD-L1 inside cells, SACC tends to secrete them out through sEVs. In the background of high plasma IFN-γ level, the low density of TIL is at least somewhat attributable to the apoptosis induced by sEV PD-L1.

In SACC patients, plasma IFN-γ level dictates the concentrations of circulating sEVs as well as the levels of PD-L1 carried by these vesicles, detected by NTA and ELISA respectively. Due to the lack of SACC animal model, we co-incubated the circulating sEVs with the primary immunocytes, and found the sEV PD-L1 promoted CD8^+^ T cell apoptosis via PD-L1/PD-1 axis. Nevertheless, the attempt to rescue this pro-apoptotic effect through blocking the PD-1 on the surface of T cells with PD-1 antibody has failed, suggesting that there may be some other proteins on sEVs, such as FasL, lead to the apoptosis of T cells.^39,40^ Also, it is noteworthy to investigate some other effects of sEV PD-L1 on CD8^+^ T cells.

In conclusion, our study shows, in a high IFN-γ circumstance, not only are SACC cells insensitive to its growth-inhibition effect, but also their migration and invasion abilities are enhanced. IFN-γ up-regulates PD-L1 synthesis and promotes sEV secretion, which carried most of the PD-L1 and induced the apoptosis of CD8^+^ T cells via PD-L1/PD-1 binding. These results indicate a mechanism that IFN-γ induces immunosuppression in SACC via sEV PD-L1, and suggest that IFN-γ insensitivity of SACC might be held accountable for the few immune cell infiltration.

## Materials and Methods

### Patients and specimen collection

All samples were collected from the participants who underwent treatments in School and Hospital of Stomatology, Wuhan University. This work was agreed by Ethics Committee of Hospital of Stomatology Wuhan University and all patients gave informed consent. Residual peripheral blood samples for preoperative blood tests were collected from SACC patients (*n* = 25) and OSCC patients (*n* = 23), followed by centrifugation at 1 550 g for 15 min at RT, twice. Plasma was collected and stored at a −80 °C refrigerator. In addition, a total of 14 SACC specimens and 17 OSCC specimens were harvested from those participants undergoing surgical treatments.

### Cell line culture

SACC-83 and SACC-LM, the human salivary adenoid cystic carcinoma cell lines, were provided by professor X.Y. Ge and S.L. Li from Peking University School and Hospital of Stomatology. After SACC-83 colonization of the lungs of immunodeficient mice, SACC-LM cells characterized by stronger lung metastatic capabilities were harvested from those metastatic lungs.^41^ SACC cells were cultured in RPMI 1640 medium (Gibco) with 10% fetal bovine serum (FBS, PAN-Biotech). CAL27 and SCC25, the human oral squamous cell carcinoma lines, were cultured in DMEM (Gibco) with 10% FBS.

### Immunohistochemistry

SACC and OSCC tissues were submerged in 4% paraformaldehyde overnight. After dehydration, tumor tissue samples were embedded in paraffin. All samples were sectioned at 4 μm. The sections were dewaxed, rehydrated and subjected to antigen retrieval by microwave heating. Then, inactivation of endogenous peroxidase in the sections was performed by incubation with 3% hydrogen peroxide. Goat serum was used to reduce nonspecific staining. Primary PD-L1 (1:200, Cell Signaling Technology) antibody was incubated with those sections for 8 h at 4 °C. After soaking with secondary antibodies for 20 min at 37 °C, primary antibodies that had bound to PD-L1 in the sections were linked to secondary antibodies. Then the sections were reacted with horseradish peroxidase-conjugated streptavidin for another 20 min. The sections were developed with DAB, followed by counterstaining with hematoxylin. Expression level of PD-L1 was calculated as IOD/area and conducted by Image Pro Plus.

### Western blot analysis

Enhanced BCA Protein Assay Kit (Beyotime) was used for determining the protein content of SCC-25, CAL27, SACC-83, SACC-LM cells and sEVs. Protein samples were fractionated on a 10% gel SDS-PAGE and then transferred to a polyvinylidene fluoride (PVDF) membrane. The PVDF membrane was soaked in 5% milk with TBST for 1 h at room temperature (RT). The bolts were then incubated with primary antibodies for 10 h at 4 °C. Primary antibody dilution was shown in Appendix Materials and Methods. After incubating with corresponding secondary antibodies (HRP-conjugated) for 1 h at RT, the blots were developed with ECL detection reagents (Sigma‑Aldrich).

### Cell viability assay

Cell Counting Kit8 (CCK8, Biosharp) were used to determine the cell proliferation. Briefly, 5 × 10^3^ of SACC-83, SACC-LM, CAL27 and SCC25 cells were seeded in 96 well plates and cultured for 48 h. Jurkat T cells were seeded at a density of 1 × 10^4^ per well and co-cultured with sEVs for 24 h. Then CCK-8 reagent was added to the plates and cultured for another 1.5 h at 37 °C. Finally, the 96-well plates were read at 450 nm wavelength. The OD values were compared and presented as the percentage (%) of control group.

### Real-time quantitative PCR

RNA was reverse transcribed to cDNA by using the HiScript II Reverse Transcriptase (Vazyme). After that, obtained cDNA was amplified on a QuantStudio 6 Flex Real-Time PCR System (Bio-Rad) through the standard procedures. All samples were amplified with forward and reverse primers. GAPDH was selected as internal control, and the results were represented as fold changes compared with controls.

### Wound-healing assay

SACC-83 cells were cultured in a 6-well plate and after reaching 80 % confluence, the cell monolayers were scratched with asterile pipette tip. Then original culture medium was removed and fresh FBS free RPMI 1640 with IFN-γ (100 ng·mL^−1^, Peprotech) was added into the plate for another 24 h at 37 °C. The wound areas were observed at regular intervals under an inverted microscopy and quantified by ImageJ. Wound sizes were compared and presented as the wound healing rate.

### Transwell chamber assay

Transwell inserts with 8 μm pore size (Corning) were used for experiment. After treating with IFN-γ (100 ng·mL^−1^) for 12 h, 1 × 10^5^ of SACC-83 cells were transferred to the upper chamber and cultured for 18 h. For invasion assay, 20 μg matrigel (Corning) were coated to the upper chamber, then 1 × 10^5^ SACC-83 cells were seeded to the coated-chamber and cultured for 18 h. Paraformaldehyde was used to fix the cells in transwell inserts. The fixed cells were washed and followed by staining with crystal violet. Crystal violet stained cells in the upper chamber were scraped, and those in the lower chamber were photographed microscopically and counted by using ImageJ software. Cell counts were compared and present as the percentage (%) of control cells.

### sEV isolation

For sEV isolation from SACC-83 cell culture supernatants, 10% exosome-free FBS was used for cell culture. Bovine EVs were removed by overnight ultracentrifugation at 120 000 g. SACC-83 cells were cultured in IFN-γ (80 ng·mL^−1^) for 48 h to harvest IFN-γ-induced sEVs. Cell culture medium was purified by a standard differential centrifugation protocol.^42,43^ Specifically, the cell pellet was first discarded by centrifuging at 500 g, and the remaining cell culture medium was further centrifuged (5810 R, Eppendorf) at 3 000 g for 20 min twice. The obtained cell culture medium was then ultracentifugated (Optima XPN-100, Beckman Coulter) at 120 000 g for 70 min at 10 °C. The sEV pellet was suspended with sterile PBS and aliquoted.

To remove debris and large EVs, plasma sample was first centrifuged (5424 R, Eppendorf) at 10 000 g for 45 min. Then, the obtained supernatant was ultracentifugated (Optima™ MAX-XP, Beckman Coulter) at 120 000 g for 70 min at 10 °C and the circulating sEV pellet was suspended with sterile PBS.

### sEV labeling and cellular uptake

Purified sEVs were stained with CFSE membrane dye (Beyotime). Specificly, a total of 100 μg sEVs were diluted in 100 μL PBS, then stained at 50 nmol·L^−1^ CFSE for 15 min at 37 °C. CFSE labeled sEVs were isolated by ultracentrifugated (Optima™ MAX-XP) at 120 000 g for 45 min at 10 °C. CellMask (Thermo Fisher Scientific) was used to label Jurkat T cells at 37 °C for 10 min. CD18 antibody (0.5 μg·mL^−1^, Biolegend) was incubated with CellMask-labeled Jurkat T cells at 37 °C for 1 h. Finally, CFSE-labeled sEVs (20 μg·mL^−1^) were co-cultured with CellMask-labeled cells for 12 h. The samples were observed under a Ti2-U inverted microscope (Nikon).

### Nano-flow cytometry

Purified sEVs were stained with PD-L1-PE (Biolegend) and CD54-FITC (Biolegend) for 1 h at 37 °C. The stained sEVs were then labeled with wheat germ agglutinin (WGA, Thermo Fisher Scientific) membrane dye for 15 min. Those sEV samples were measured by a micro plus flow cytometry (Apogee). The data was calculated by FlowJo X.0.7 software.

### Measurement of cell apoptosis

SACC cells were obtained after IFN-γ treatment (10 ng·mL^−1^) for 24 h. PD-1 on Jurkat T cell was blocked with PD-1 antibody (10 μg·mL^−1^, sintilimab, Innovent) for 1 h, followed by incubation with IFN-γ-induced sEVs (20 μg·mL^−1^) for 12 h. To harvest primary immunocytes, human cervical lymph nodes were milled and filtered with a 70 μm strainer, followed by erythrocyte lysate treatment. Similarly, after blocking surface PD-1 with PD-1 antibody (10 μg·mL^−1^, sintilimab, Innovent), the immunocytes were incubated with IFN-γ-induced sEVs (30 μg·mL^−1^) and circulating sEVs (30 μg·mL^−1^) for 12 h.

Harvested cell samples were suspended using 50 μL of 1× binding buffer, followed by incubatation with Annexin V-PerCP-Cy5.5 (BD Biosciences) antibody for 15 min at RT. Before flow cytometry analysis, 400 μL of 1× binding buffer was used to diulte each sample and the assay was completed within 1 h.

### ELISA

For detection of IFN-γ, the plasma samples were determined according to the instruction manuals of human IFN-γ ELISA kit (Mabtech). PD-L1 on sEVs in SACC-83 supernatants and patients’ plasma were measured as previously described.^22^ Briefly, ELISA 96-well plate was coated with 0.25 μg per well of PD-L1 antibody (clone 5H1, Millipore) for 12 h at 4 °C. The standard curve was made with recombinant human PD-L1 protein. Then, 100 μL (15 μg) of SACC-83 sEVs or 100 μL (100 μg) of circulating sEVs were added to PD-L1-coated plate and incubated for 12 h at 4 °C. The detection antibody (biotinylated PD-L1, clone MIH1, eBioscience) was diluted in PBS and added to the plate. After incubation with the detection antibody for 1 h, streptavidin-HRP (Mabtech) was diluted 1:1 000 and added to each well. Finally, the plates were developed with TMB for 10 min followed by neutralization with H_2_SO_4_ and examined at 450 nm and 570 nm.

### Characterization of sEVs

For characterization of the purified sEVs by using transmission electron microscopy (TEM), purified sEVs were dropped on a copper grid for 5 min. Then the copper grids were washed with PBS 3 times, followed by incubating with 1% v/v uranyl acetate for 40 s. Then copper grids were observed on a HT7700 TEM (Hitachi). The diameter and particle number of purified sEVs were determined by ZetaView nanoparticle tracking analyzer (NTA, Particle Metrix).

### Statistical Analysis

All data in this study were presented as mean ± S.D. Student’s t-test was conducted to analysis the differences between two groups, while one-way ANOVA was conducted for comparison of multiple groups. Comparison of cell growth curves of different groups were performed by two-way ANOVA. Correlations between different variables were analyzed through spearman test. The significance of difference of nominal variables was analyzed by fisher exact test. Statistical analysis was conducted by Prism 7 software, and *p* value < 0.05 was considered statistically significant.

## Supplementary information

Supplementary Material
