# Supplementary material for: Interferon-γ induces immunosuppression in salivary adenoid cystic carcinoma by regulating programmed death ligand 1 secretion
Source: Int J Oral Sci. 2022 Sep 28;14:47. doi: 10.1038/s41368-022-00197-x (PMC9515071; doi:10.1038/s41368-022-00197-x)
Supplement: Supplementary file 1 — Supplementary Material [file 41368_2022_197_MOESM1_ESM.docx]

**Title: IFN-γ induces immunosuppression in salivary adenoid cystic carcinoma by regulating PD-L1 secretion**

Running title: IFN-γ induces immunosuppression in SACC

Qiu-Yun Fu^1^, Xing-Chi Liu^1^, Hou-Fu Xia^1,2^, Yi-Cun Li^3^, Zi-Li Yu^1,2^, Bing Liu^1,2^, Xue-Peng Xiong^1,2*^, Gang Chen^1,2,4*^

^1^The State Key Laboratory Breeding Base of Basic Science of Stomatology & Key Laboratory of Oral Biomedicine Ministry of Education, School and Hospital of Stomatology, Wuhan University, Wuhan 430079, China.

^2^Department of Oral and Maxillofacial Surgery, School and Hospital of Stomatology, Wuhan University, Wuhan 430079, China.

^3^Peking University Shenzhen Hospital, Shenzhen Peking University-The Hong Kong University of Science and Technology Medical Center, Shenzhen 518000, China.

^4^Frontier Science Center for Immunology and Metabolism, Wuhan University, Wuhan 430071, China.

***Correspondence Authors**

Xue-Peng Xiong, MD, Associate professor, Email: xiongxuepeng@whu.edu.cn,

Gang Chen, MD, Professor, Email: geraldchan@whu.edu.cn. Tel: +86 27 87686125; Fax: +86 27 87873260.

**Appendix Materials and Methods**

Western Blot Analysis

Primary antibody dilutions were as follows: β-Actin (1:5 000, ABclonal Technology), PD-L1 (1:1 000, Cell Signaling Technology), E-cadherin (1:1 000, Abcam), GAPDH (1:5 000, ABclonal Technology), Stat1 (1:1 000, Cell Signaling Technology), and p-Stat1 (1:500, Cell Signaling Technology), Vimentin (1:1 000, Cell Signaling Technology), MMP9 (N-terminal, 1:1 000, Proteintech), CD9 (1:1 000, Cell Signaling Technology), GM130 (1:1 000, Cell Signaling Technology), Alix (1:1 000, Abcam), Rab27a (1:1 000, Cell Signaling Technology), TSG101 (1:1 000, Abcam), Hrs (1:1 000, Cell Signaling Technology), Slug (1:1 000, Cell Signaling Technology).

**Appendix Table 1. Demographic data and clinicopathological features of the samples.**

|  | Variable | Number (%) |  | Variable | Number (%) |
| --- | --- | --- | --- | --- | --- |
| SACC | Gender |  | OSCC | Gender |  |
|  | Male | 7 (28) |  | Male | 6 (26.1) |
|  | Female | 18 (72) |  | Female | 17 (73.9) |
|  | Age |  |  | Age |  |
|  | 20-39 | 4 (16) |  | 30-39 | 4 (17.4) |
|  | 40-49 | 5 (20) |  | 40-49 | 2 (8.7) |
|  | 50-59 | 8 (32) |  | 50-59 | 6 (26.1) |
|  | 60-69 | 5 (20) |  | 60-69 | 9 (39.1) |
|  | 70-79 | 3 (12) |  | 70-89 | 2 (8.7) |
|  | Primary tumor (T)* |  |  | Primary tumor (T)* |  |
|  | T1 and T2 | 12 (50) |  | T1 and T2 | 13 (61.9) |
|  | T3 and T4 | 12 (50) |  | T3 and T 4 | 8 (38.1) |
|  | Regional lymph node involvement (N) * |  |  | Regional lymph node involvement (N) * |  |
|  | N0 | 21 (87.5) |  | N0 | 11 (52.4) |
|  | N1 | 3 (12.5) |  | N1 | 10 (47.6) |
|  | Lung metastasis (M) *^#^ |  |  | Lung metastasis (M) * |  |
|  | M0 | 18 (75) |  | M0 | 21 (100) |
|  | M1 | 6 (25) |  | M1 | 0 |
|  | Anatomic location |  |  |  |  |
|  | Minor salivary gland |  |  |  |  |
|  | Palate | 10 (40) |  |  |  |
|  | Tongue | 4 (16) |  |  |  |
|  | Buccal mucosa | 1 (4) |  |  |  |
|  | Major salivary gland |  |  |  |  |
|  | Sublingual | 4 (16) |  |  |  |
|  | Submandibular | 4 (16) |  |  |  |
|  | Parotid | 2 (8) |  |  |  |

* Based on the American Joint Committee on Cancer (AJCC, 8th Edition).

# Lung metastasis was not available for one SACC patient.

TNM stage was not available for one SACC patient and two OSCC patients.

**Appendix Table 2. Relationship between the clinicopathological data of SACC patients and plasma IFN-γ level.**

| Variable | Total | Plasma IFN-γ | | *P*-value |
| --- | --- | --- | --- | --- |
|  |  | Low | High |  |
| Gender |  |  |  |  |
| Male | 7 | 2 | 5 | 1 |
| Female | 18 | 6 | 12 |  |
| Age |  |  |  |  |
| ≤54 | 13 | 4 | 9 | 1 |
| >54 | 12 | 4 | 8 |  |
| Anatomic location |  |  |  |  |
| Minor salivary gland | 15 | 3 | 12 | 0.193 6 |
| Major salivary gland | 10 | 5 | 5 |  |
| Primary tumor (T)* |  |  |  |  |
| T1 and T2 | 12 | 2 | 10 | 0.193 0 |
| T3 and T4 | 12 | 6 | 6 |  |
| Regional lymph node involvement (N) * |  |  |  |  |
| N0 | 21 | 5 | 16 | 0.027 7 |
| N1 | 3 | 3 | 0 |  |
| Lung metastasis (M) *^#^ |  |  |  |  |
| M0 | 15 | 8 | 10 | 0.066 4 |
| M1 | 6 | 0 | 6 |  |

* Based on the American Joint Committee on Cancer (AJCC, 8th Edition).

# Lung metastasis was not available for one patient.

Undetectable plasma IFN-γ was determined as low level.

Detectable plasma IFN-γ was determined as high level.

TNM stage was not available for one patient.

Statistical analyses were performed using the Fisher’s exact test.


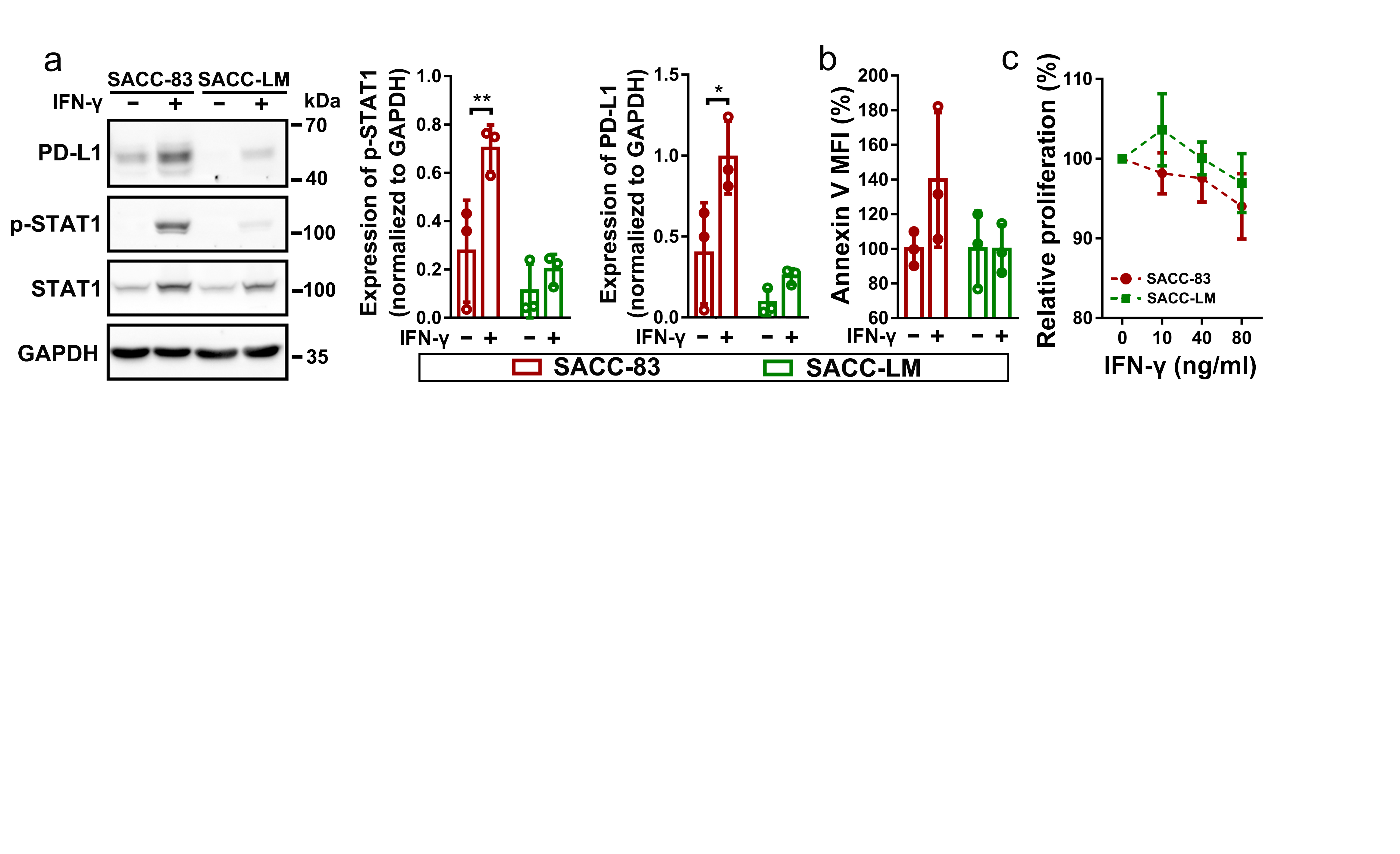


**Appendix Figure 1. Comparison of the sensitivities of SACC-83 and SACC-LM to IFN-γ.** **a** Western blot analysis of IFN-γ/STAT1/PD-L1 pathway on SACC-83 and SACC-LM with or without IFN-γ treatment (left). Quantification of the expression of p-STAT1 and PD-L1 (middle and right, normalized to GAPDH) (n = 3). **b** Flow cytometry analysis of the expression level of Annexin V on SACC-83 and SACC-LM with or without IFN-γ treatment (n = 3). **c** Effect of IFN-γ on proliferation of SACC-83 and SACC-LM determined by CCK8 assay (n=3). *, *P* < 0.05; **, *P* < 0.01.

**Appendix Figure 2. Gating strategies and the expression levels of sEV PD-L1 and CD54.** **a** Representative plots of size distribution of standard beads, (polystyrene standard beads, Si: silicon standard beads), and representative contour plots showing the gating strategies were used to identify the PD-L1^+^CD54^+^ sEVs. **b, c** Comparisons of mean fluorescence intensity (MFI) of PD-L1 or CD54 between control sEVs (C) and IFN-γ-induced sEVs (IFN-γ) respectively (n = 3). *, *P* < 0.05; ***, *P* < 0.001.


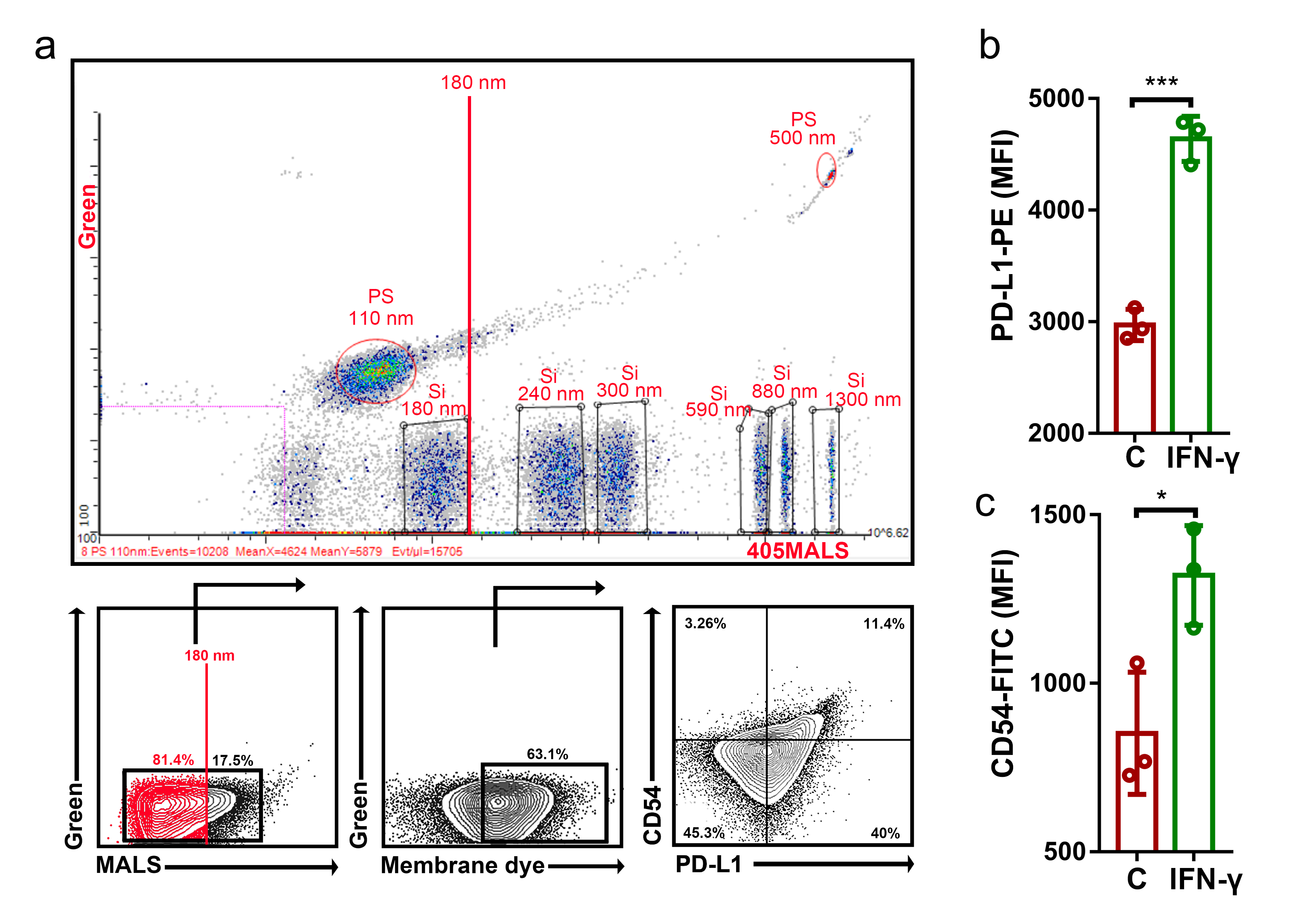


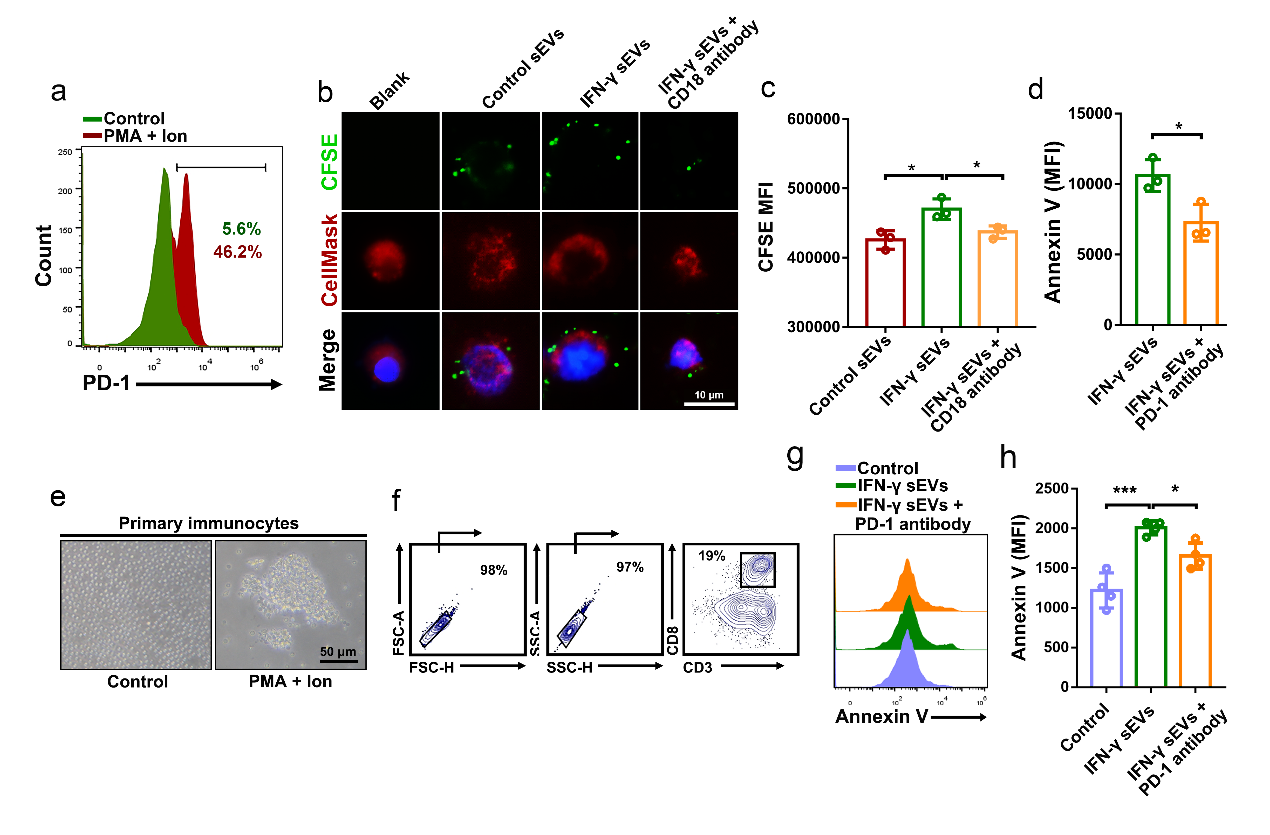


**Appendix Figure 3.** **The binding and apoptosis effect of IFN-γ-induced sEVs derived from SACC-83 on T cells.** **a** Representative histogram of Jurkat T cells after treatment with PMA + Ion for 36 h. **b** Immunofluorescence staining of CellMask-labeled Jurkat T cells (stimulated with PMA + Ion) after treatment with CFSE-labeled control sEVs and IFN-γ-induced sEVs with or without anti-CD18 antibody blocking. Scale bar: 10 μm. **c** The MFI of CFSE on Jurkat T cells after incubated with CFSE-labeled control sEVs and IFN-γ-induced sEVs with or without anti-CD18 antibody blocking (n = 3). **d** The MFI of Annexin V of Jurkat T cells after incubating with IFN-γ-induced sEVs with or without anti-PD-1 antibody blocking (n = 3). **e** Representative images showing the clustering of immunocytes after PMA + Ion treatment. Scale bar: 50 μm. **f** Gating strategy for identifying the CD3^+^CD8^+^ T cells. **g** Representative histogram of CD8^+^ T cells with PMA + Ion stimulation after incubating with IFN-γ-induced sEVs for 12 h. **h** The MFI of Annexin V of CD8^+^ T cells after incubating with IFN-γ-induced sEVs with or without anti PD-1antibody blocking (n = 4).*, *P* < 0.05; ***, *P* < 0.001.


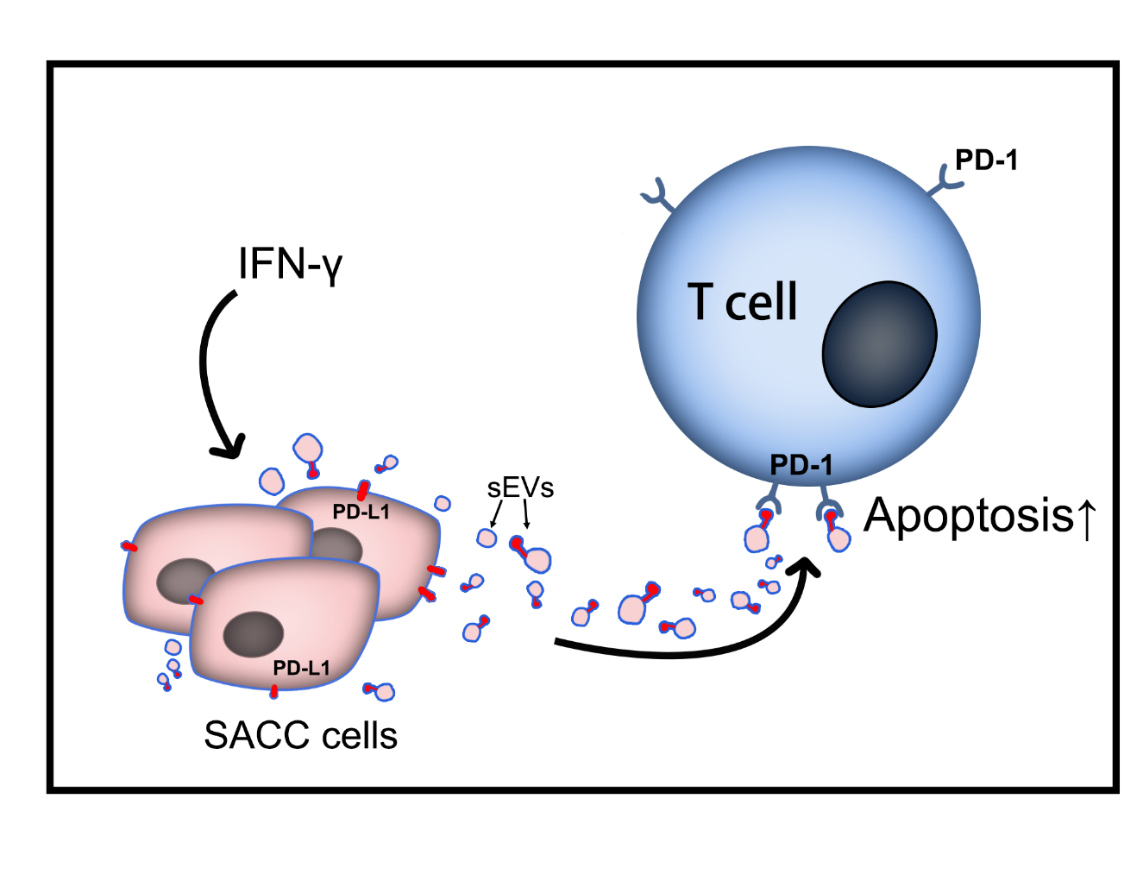


**Appendix Figure 4. Diagram of immunosuppression in SACC induced by IFN-γ.** IFN-γ up-regulates PD-L1 expression and promotes the secretion of sEVs, which carry most of the PD-L1, and induces the apoptosis of CD8^+^ T cells through PD-L1/PD-1 binding.
